# Knowledge and Practice of Herbal Medicine on Oral Health among Dental Personnel in Malaysia

**DOI:** 10.12688/f1000research.129865.1

**Published:** 2023-03-14

**Authors:** Abedelmalek KalefhTabnjh, Wan Muhamad Amir W Ahmad, Ruhaya Hasan, Mohd Zulkarnain bin Sinor, Mauro Henrique Nogueira Guimarães de Abreu, Ling Shing Wong, Sinouvassane Djearamane, Siddharthan Selvaraj

**Affiliations:** 1 School of Dental Sciences, Universiti Sains Malaysia (USM), Kubang Kerian, Kota Bharu, Kelantan, 16150, Malaysia; 2 Department of Applied Dental Sciences, Jordan University of Science and Technology, Irbid, 22110, Jordan; 3 School of Dentistry, Universidade Federal de Minas Gerais, Belo Horizonte, 31270-901, Brazil; 4 Faculty of Health and Life Sciences, INTI International University, Nilai, 71800, Malaysia; 5 Department of Biomedical Science, Faculty of Science, Universiti Tunku Abdul Rahman, Kampar, Perak, 31900, Malaysia; 6 Faculty of Dentistry, AIMST University, Bedong, 08100, Malaysia

**Keywords:** Alternative medicine, Herbal medicine, Knowledge, Practice, Oral health

## Abstract

Objective: To investigate the association between knowledge and practice of medical herbs related to oral heath among staffs at School of Dental Sciences, Universiti Sains Malaysia,Malaysia. Methods: A cross-sectional study was conducted at the School of Dental Sciences, USM, on 100 lecturers, dental surgery assistants, nurses, administrative workers, and dental technicians. A set of knowledge and practice questionnaire was used in this study. Descriptive statistics, chi **-**square and correlation were used to analyze the data. Results: The results showed that the dental staff’s knowledge of oral herbal medicines was 32.8%. This indicates that they had a moderate level of knowledge. Almost three-quarters of the employees (73%) practiced at a low level (0-25%), while only 13.85% practiced at a moderate level. The findings revealed that there was no significant relationship between knowledge and practice scores and age or gender. The results, from the other hand, revealed a significant association between practice scores and the type of occupations among staff (P=0.04). The Correlation analyses shows a moderately positive relationship between knowledge and practice. Conclusions: Dental staff had a moderate level of knowledge and a poor level of practice with regard to herbal medicines for oral health. There was a significant association between age and knowledge and there was a significant association between the knowledge and practice among the dental personnel

## Introduction

According to the World Health Organization (WHO) in 2000, traditional medicine is “a sum total of the knowledge, skills and practices based on theories, beliefs and experience indigenous to different cultures, whether explicable or not, used in the maintenance of health as well as prevention, diagnosis, improvement or treatment of physical and mental illness”. For thousands of years, humans have used herbs and herbal products as an important part of traditional and alternative medicine. ^1^

Herbal medicine has developed into an intriguing field of study for scholars world-wide during the last few decades. ^1^ Over 30% of plant species are utilized in medical treatments due to their compounds, which have beneficial long-term impacts on human health, particularly in the treatment of diseases. ^2^ According to WHO (2000), traditional medicine is used by half of the population in developed countries and by about 80% of the people in developing countries. In addition, over 25% of medicines are based on herbs and herbs. ^1^^,^^3^

Problems in the oral cavity can have a significant impact on persons’ general well-being by causing significant pain and suffering, lowering their quality of life. ^4^^,^^5^ Caries and periodontal diseases are the most common mouth issues worldwide as a result of this issue. ^6^ Caries was found in 85 percent of children, 75 percent of adolescents, and 98 percent of adults worldwide. In the same setting, the prevalence of severe periodontal disease, which results in tooth loss, is 15-20% in the same age group. ^7^

Kim and Lean in the year 2013, conducted a study at the Hospital Universiti Sains Malaysia in Kelantan, Malaysia, to investigate the level of knowledge and practice of 460 pregnant women regarding herbal medications. It was observed that the majority of women (89.8%) had limited knowledge of herbs. Only 8.5% of women stated that they were aware of the components in their herbal medications. However, none of them were able to identify the active compounds in the herbal medications they were taking. Only 11.5% of survey participants were aware that herbal medicine can be contaminated with bacteria, mercury, or contaminants. ^8^

Often people obtain information about herbal medicine from friends and family rather than from health care providers. This was supported by a study on the use of alternative medicines by cancer patients in Malaysia ^9^ and another on the use of alternative medicines by community members with chronic kidney disease (CKD) in northern Tanzania. This means that they respect the opinion of family members, elders, friends, traditional healers, and herbal vendors more highly. ^10^

A study conducted in Cameroon on the herbs and plants used by traditional healers to address oral health concerns showed that the people relied on 52 plants, including onion, sweet potato, and coconut. The most often prescribed medications included those for toothache, mouth sores, sore throat, mouth ulcers, abscess, bullous lesion, broken tooth, mouth thrush, dentine sensitivity, dental caries, gingivitis, tonsillitis, sinusitis, dry mouth, oral syphilis, dental extraction, and oral cancer. Common plant components such as roots, leaves, and bark are utilized to treat dental disorders. ^11^

In Malaysia, most Malaysians agree that herbal products are fully safe, have no dangerous or harmful chemicals and have no adverse effects on other commercial pharmaceutical medications. ^8^ The majority relied on observations, experiences, and rituals de-rived from beliefs and cultures passed down through generations. ^8^ According to Yusof *et al.*, 2007, more than half of Malaysians who participated in the study (56.8%) used alternative medicine to relieve orofacial pain. ^12^

The goal of this study was to examine the level of knowledge and practice of dental staff at the USM Health Campus’s Dental School regarding medical herbs and plants relevant to oral health. Additionally, the aim was to determine whether there is a relationship between knowledge and practice of using herbs as medicine associated with oral health and sociodemographic characteristics.

## Materials and methods

This cross-sectional study was conducted at the School of Dental Sciences, USM, in Kelantan state, Malaysia in the period between February 2018 to July 2018.

### Participants

The whole dental staff at USM’s School of Dental Sciences was recognized as the study’s reference population. Lecturers, DSAs (Dental Surgical Assistants), nurses, administrative personnel, and dental technicians were among those who responded. Only participants who are USM employees, have Malaysian nationality, and are between the ages of 18 and 60 were chosen. Anyone over the age of 60, as well as non-USM employees, were excluded from this study. All respondents who met the inclusion criteria were sampled using both probability stratified and simple random sampling methods to minimize the bias in the study. Nurses, dental technicians, administration workers, DSAs, and lecturers made up the dental personnel, split into occupational (strata) categories. The strata were then sampled using simple random sampling. Following that, subjects were chosen as respondents for this study from each stratum based on the workers’ ratio to the total number of dental staff. There were 30 nurses, 15 dental technicians, 16 administrator workers, 16 DSA, and 30 lecturers among those who participated. Sample size was calculated according to study carried out by Farooqui and team in the year 2016 ^9^ and it was 91, and by adding 10% drop out the total sample size was 101. Sample size was calculated using Arifin WN. Sample size calculator (Version 2.0). The data underlying the results and the questionnaire used can be found in Figshare. ^28^

### Data collection and tools

The tool for this study was a knowledge and practice questionnaire adapted from a study done by Azriani, 2007, ^13^ about the use of herbal medicines during pregnancy, then modified to be suitable for oral health. The modifications include eliminating the questions related to pregnant women which are not related to this study and adding questions more related to oral health. The dependent variables in this study were knowledge and practice of herbal medicines; the independent variables were sociodemographic characteristics. The questionnaire was validated through a validation process. Content validity was carried out and a group discussion with experts included a dental public health specialist, nutrition specialist, and a dentist to be sure that the content is clear, suitable and covering the topics. The comments and suggestions from the expert discussion group were reviewed to validate the content of the questionnaire. During face validation session, ten Malay people were asked to answer the questions and their feedback was taken about unclear points. After that the questionnaire was piloted among 30 subjects from the dental staff to be sure that it’s clear, easy to understand, and there are no misunderstandings. Minor corrections were done to the questionnaire by including ‘No information’ to the part (2) Question (8), and adding ‘If no The questions finished’ after Question (12) because one of the respondents wrongly continued to answer the next questions while he answered that he never used herbs for oral health. ^13^

The self-administered questionnaire is divided into three sections: the socio-demographic profile, knowledge of herbal medicines linked to oral health, and practice of herbal medicines connected to oral health. Subjects were asked to select the appropriate answers for sex, ethnicity, education level, married status, and occupation for socio-demographic data (close-ended questions). In terms of the subjects’ age and monthly personal income, they were asked to specify the answer (open-ended questions).

The knowledge section included five questions about herbal medicine knowledge related to oral health. The questions were about the source of information about herbs, whether or not individuals know herbs that are used for oral health, the names of herbs individuals know, how to use herbs, and the effective ingredients. Five questions were included in the practice section. These were: using herbal toothpaste, having previously used herbs, the herbs used, frequency of intake, and reason for taking herbs.

For scoring reasons, a ‘1’ was assigned to each correct response in the knowledge domain, while a ‘0’ was assigned to each incorrect response. A ‘1’ was added to the herb names to indicate that the herb was identified by the subject. ^14^ The knowledge section’s overall score range was ‘0 – 10’. In the practice domain, a ‘0’ was assigned to did not use and was unsure, but a ‘1’ was assigned to the yes choice. For the herbs used, a ‘1’ was assigned to each herb that was consumed by subjects. ^14^ For each decision made by the subject, the option ‘never’ received a score of ‘0’, ‘if necessary’, ‘every week’, and ‘every day’ received scores of “1”, “2”, and “3”, respectively, while ‘every day’ indicates the highest frequency. ^15^ A ‘1’ was assigned to each oral problem chosen by the subject in the use domain. ^14^ Knowledge and practice scores were categorized as Poor (0–25%), Moderate (26%–50%), Good (51–75%), and Very Good (76–100%) based on the proportion of total marks received for correct answers. ^16^

### Data analysis

When analyzing the data, IBM SPSS version 22.0 was used. For continuous numerical data (age and income), descriptive analysis such as mean and standard deviation was utilized, whereas frequency with percentage was used for categorical variables (sex, ethnicity, marital status, education level, and occupation). The Shapiro test was used to test the normality of the continued data. After dividing knowledge and practice scores of the participants into four groups, the Chi-square test and Fisher exact test were used to examine the associations between herbal medicine knowledge and practice groups and socio-demographic factors. A Pearson correlation test was used to explore the relationship between knowledge and practice scores.

### Ethical consent

The Human Research and Ethics Committee of Universiti Sains Malaysia granted ethical approval (USM /JEPeM/17120725). Additionally, the researcher explained the study to participants who decided to participate and got a formal consent form to ensure that they agreed voluntarily to participate. The data were kept confidential and informed written consent was received from the participants who were kept anonymous.

## Results

A total of 107 respondents were invited to participate in this study; however, 100 respondents completed the study, giving a 93% response rate. The majority (98%) were Malay, while the remaining 2% were Chinese. 70% of participants were female, whereas 30% were male. The participants’ mean (SD) age was 37.3 (7.4) years, ranging from 23 to 58 years ( Table 1). There was no statistically significant difference in mean ages between males and females (p = 0.278). There were 17 participants who didn’t answer the question regarding the income and 4 participants didn’t answer the age question. These cases were considered as missing data while testing the association with income and age variables and were therefore excluded.

**Table 1.** Socio-demographic characteristics of the dental staff (n = 100).

Variables	*n*(%)
**Sex**	
Male	30(30)
Female	70(70)
**Ethnicity**	
Malay	98(98)
Chinese	1(2)
**Age** ^a^	37.3(7.4)
**Personal monthly income** (RM) ^b^	3,000(4,000)
**Social status**	
Married	83(83)
Single	16(16)
Separated	1(1)
**Education level**	
SPM/Certificate	35(35)
Diploma	30(30)
Degree	8(8)
Master	12(12)
PhD	15(15)
**Occupation**	
Lecturer	28(28)
Admin.	15(15)
Nurse	15(15)
Dental Technician	14(14)
Dental Surgical Assistant	28(28)

^**a**^ Mean (SD).

^**b**^ Median (IQR).

### Knowledge about herbal medicine related to oral health

More than one-third of the staff (35%) had a poor level of knowledge; another (48%) had a moderate level of knowledge, only (14%) had a good level of knowledge, and (3%) had a very good level of knowledge. The mean (SD) of knowledge score (Ks) was 3.3 (2.4), indicating that 32.8 % of dental staff were knowledgeable about herbal medicines related to oral health. This shows that they had a moderate level of knowledge of the topic. Table 2 shows the respondents’ knowledge categories.

**Table 2.** Knowledge categories (n = 100).

Knowledge categories		*n*(%)
a. Mean(SD) ^a^		3.3(2.4)
b. Poor	(0–25)%	35(35)
Moderate	(26–50)%	48(48)
Good	(51–75)%	14(14)
Very good	(76–100)%	3(3)

^a^ Knowledge scores.

Table 3 shows respondents’ answers to questions about their knowledge of herbal medicines related to oral health. The internet was the primary source of information about herbal medicines for (65%). Approximately one-third (29%) of those respondents were reliant on their friends for knowledge regarding herbal medicines.

**Table 3.** Knowledge about herbal medicines related to oral health (n = 100).

Items	*n*(%)
**Source of information**	
No information	2(2)
Own self	18(18)
Sibling	9(9)
Friends	29(29)
Parents	15(15)
Traditional healers	5(5)
Clinic/hospital staff	22(22)
Husband/wife	8(8)
Internet	65(65)
Others	3(3)
**Know herbs that are used for oral health**	
Yes	57(57)
No	8(8)
Not sure	35(35)
**Names of herbs**	
Miswak	66(66)
Cinnamon	18(18)
Cloves	60(60)
Aloe Vera	9(9)
Garlic	16(16)
Mentha	42(42)
Coconut roots	-
Others	3(3)
**Know how to use**	
Yes	36(36)
No	23(23)
Not sure	41(41)
**Know the effective ingredients**	
Yes	24(24)
No	20(20)
Not sure	56(56)

When asked if they were familiar with any herbs used for dental health, moreover half (57%) responded yes. Miswak was once the most well-known oral health herb. 66% of respondents who said yes were aware it was used for oral health. Clove was the second most chosen herb, with 60% of respondents recognizing its oral health benefits. Mentha was named as one of the best oral health herbs by 42% of respondents.

More than half of the workers (64%) have no idea how to use herbs for oral health and only about a quarter of them were aware of the herbs’ active ingredients. Even among those who responded that they knew of the use of herbs for oral health, only 56% knew how to use them, and only 35% knew the effective ingredients.

### Practice about herbal medicines related to oral health

Almost three-quarters of the staff (73%) were in poor practice (0-25%), another 21% were in a moderate level of practice (26-50%), only 6% were in a good level of practice (51-75%), and none were in a very good level of practice (76-100%). The mean (SD) of the practice score (Ps) was 2.7 (3.95), indicating that there was a low level of practice among dental personnel when it came to use of herbal medicines for oral health. Table 4 shows the respondents’ categories of practices.

**Table 4.** Practice categories (n = 100).

Practice categories		*n*(%)
a. Mean (SD) ^a^		2.7(3.95)
b. Poor	(0–25)%	73(73)
Moderate	(26–50)%	21(21)
Good	(51–75)%	6(6)
Very good	(76–100)%	-

^a^ Practice scores.

According to the findings, only 25% of the staff used herbal toothpaste. Only one-third of respondents said they utilized herbs for dental health (34%). Miswak was used by the majority of responders (82.4%), whereas Mentha was used by about 52.9%. Clove was the third most popular herb, accounting for 38.2% of all uses. In terms of frequency of use, 44.1% used it only when necessary, 5.9% once per week, and 41.2% once per day. When asked why they used herbs for oral health, 70.6% said it was for oral hygiene, 38.2% for pain relief, 38.2% for teeth whitening, and 29.4% for bad breath. Table 5 shows results for the practice of using herbal medicines.

**Table 5.** Practices toward herbal medicines related to oral health (n = 100).

Items	*n*(%)
**Using herbal toothpaste**	
Yes	25(25)
No	64(64)
Not sure	11(11)
**Using herbs in the past**	
Yes	34(34)
No	66(66)
**If yes**	
**herbs used**	
Miswak	28(82.4)
Cinnamon	6(17.6)
Cloves	13(38.2)
Aloe Vera	4(11.8)
Garlic	4(11.8)
Mentha	18(52.9)
Others	1(2.9)
**Frequency of taking**	
Every day	14(41.2)
Every week	2(5.9)
Only if necessary	15(44.1)
**Purpose of using herbs**	
Oral hygiene	24(70.6)
Bad breath smell	10(29.4)
Relieve teeth pain	13(38.2)
Gingival bleeding	4(11.8)
Teeth whitening	13(38.2)
Gingival enlargement	6(17.6)
Others	1(5.9)

### Association between knowledge and practice with socio-demographic variables

The association between knowledge and age showed a significant relationship between knowledge score and age group at the 95% confidence level. Older respondents have a higher knowledge score than younger ones, χ ^2^ = 9.429 (p = 0.018). There was no association between knowledge score and sex. These findings are shown in Table 6.

**Table 6.** Association between knowledge with age and sex (n = 100).

	Knowledge level *n*(%)				χ ^2^ (df)	p-Value
	Poor 0-25	Moderate 26-50	Good 51-75	V. Good 76-100		
**Sex**						
Male	11(36.7)	14(46.7)	4(13.3)	1(3.3)		
Female	24(34.3)	34(48.6)	10(14.3)	2(2.9)	0.377(3)	1.000 ^a^
**Age group**						
≤40	30(44.1)	28(41.2)	9(13.2)	1(1.5)		
>40	4(14.3)	17(60.7)	5(17.9)	2(7.1)	9.429(3)	0.018 ^a^

^a^ Fisher exact test.

There is also no significant association of 95% confidence level between practice scores with age and sex. The value of fisher exact test for sex with practice score was χ ^2^ = 1.358 (p = 0.543), and for practice score with age it was χ ^2^ = 1.623 ( *p* = 0.454). These findings are shown in Table 7.

**Table 7.** Association between practice with age and sex (n = 100).

	Practice score *n*(%)			χ ^2^ (df)	p-Value
	Poor 0-25	Moderate 26-50	Good 51-75		
**Sex**					
Male	21(70)	6(20)	3(10)		
Female	52(74.3)	15(21.4)	3(4.3)	1.358(2)	0.543 ^a^
**Age group**					
≤40	51(75.0)	12(17.6)	5(7.4)		
>40	19(67.9)	8(28.6)	1(3.6)	1.623(2)	0.454 ^a^

^a^ Fisher exact test.

The result of the association between knowledge scores and respondents’ occupations showed that there was no association (p = 0.227). On the other hand, the results showed a relationship between practical scores and the respondents’ occupations at 95% confidence ( *p* = 0.048). Dental nurses had the highest practice level (18.3%) by occupation, followed by administrative workers (18.2 %). Table 8 shows these findings.

**Table 8.** Level of knowledge and practice for occupational groups (n = 100).

Occupations	Knowledge Score (%) (p value = 0.227 ^a^)	Practice Score (%) (p = .048 ^a^)
Lecturer	25.9%	14.3%
Admin.	17.9%	18.2%
Nurse	31.7%	18.3%
Dental	29.7	10.3%
Technician	21.6	10.7%
Dental Surgical Assistant		

^a^ Fisher exact test.

At a 95% confidence level, there was a significant association between knowledge and practice score groups (p < 0.001). According to the correlation test, there was a significant positive moderate degree correlation between these two variables (p = 0.001) (r = 0.449). As a result, participants with a high degree of knowledge also have a high level of practice.

## Discussion

To our knowledge, this was the first study to examine the knowledge and practice of Malaysians in general, and dental personnel in particular about herbal medicine for oral health. According to the respondents’ ethnic origins, 98% were Malay, and only 2% were Chinese. This may be explained by the fact that the School of Dental Sciences at USM is located in the state of Kelantan, where the majority of the population is Malay. ^17^

Most dental personnel depend heavily on the internet for information about herbal medicines (65%). This finding contrasts with studies done by Azriani ^13^ and Kim & Lean ^8^ on Malaysian pregnant women’s knowledge and practice of herbal medications, which discovered that the primary source of information was their parents. While Yeong and Choong in the year 2017, conducted a study on the knowledge and features of herbal supplement use among a Malaysian population, they discovered that only 13.3% obtained information from the internet. The disparate sources could be explained by the natures of oral disorders, pregnancy, and other ailments encountered by the population. Additionally, the internet source has gained popularity in recent years due to its ease of access to all locations. ^18^

The current study found that 24% of workers were aware of the active components in herbs, compared to 8.5%% in Kim & Lean’s study. ^8^ The larger rate could be explained by the educational disparity between the two surveys. In Kim & Lean’s study, most women only attended secondary school, whereas respondents in this survey have a greater education degree. In terms of how to use herbs, both studies found that the majority of participants were unaware of how to do so, but if we consider only those who used herbs in this study, most of them knew how to use it. The participants had a medium level of knowledge. However, contrary to other studies, most Malaysians had a poor level of understanding of herbal treatments. The disparity in outcomes could be attributed to the participants’ high level of education. ^8^

In comparison to numerous previous studies evaluating physicians, ^19^ medical practitioners, ^20^ and other healthcare professionals such as pharmacists, ^21^ and nurses, the findings of this study revealed that dental staff had a higher level of knowledge about herbal medicine. ^22^^,^^23^ The disparity could be because this study solely tested for knowledge on herbal medicine related to oral health. In comparison, the other research examined participants’ knowledge of various types of herbal medicine.

The majority of individuals did not use herbal toothpaste. Only 25% indicate that they have used it. This could be contributed to their awareness of the need for fluoridated toothpaste, as some individuals may believe that herbal toothpaste is not fluoridated. However, some herbal kinds of toothpaste on the market, such as Colgate ^®^ Herbal and Parodontax ^®^, are fluoridated. ^24^

This research found that dental professionals use herbal medicine for oral health at a low rate (Ps = 13.85%), which is much lower than previous studies on herbal medicine use in Malaysia. ^8^^,^^9^^,^^13^^,^^18^ Other trials ranged from 34.3%to 51.4%, which is three to four times the practice score for this study. The disparity in practice percentages may be explained by the fact that all other research focused on the use of herbs during pregnancy, cancer treatment, or overall health. Despite this, no examination has been conducted into oral health practices in Malaysia. Additionally, the samples were diverse because no investigation on the use of herbs by dental staff was conducted. Additionally, dental personnel have easy access to dental services, eliminating the need for alternative medications such as herbs for oral health.

According to this study, the dental staff utilized Miswak the most frequently for oral health. 87.5% of Miswak users did so for oral hygiene purposes. Tubaishat, 2005, ^25^ discovered consistent results in a study on the usage of Miswak for oral hygiene among the Jordanian population. This popularity can be attributed to Miswak’s shown usefulness in improving dental health and to religious views in Islamic countries regarding Miswak’s oral health benefits, as the prophet Mohammad commanded Muslims to use it five times daily. ^26^

The research showed that there was no significant relationship between dental staff knowledge and practice and sex, even though Farooqui *et al.* (2016) discovered a relationship between herb use and sex. The disparity in results could be attributed to the fact that Farooqui *et al*. ^9^ examined herbal use for cancer treatment, not oral health. However, this study’s findings were consistent with Tubaishat, ^25^ who found no correlation between herb use and sex. However, there was an association between respondents’ knowledge and age, which contradicted the research by Tubaishat and his colleagues, since they found no significant association between them. ^25^ This contradiction may refer to the difference in the herbs used in the two types of research, as Tubaishat’s research focused exclusively on Miswak. This herb has been used for generations, whereas this research includes a wide range of herbs and some of them are not used worldwide and not as popular as Miswak. The absence of a relationship between practice and age, while the existence of one between knowledge and age, may reflect dental staff’s easy access to dental care services. In any case, Kim & Lean’s ^8^ study discovered an association between herb use and age.

This study replicated the findings of *Farooqui et al.* ^9^ in terms of the association between practice scores and occupations, with both studies indicating a significant association. However, their findings regarding the relationship between knowledge score and professions were contradictory. Farooqui *et al.* discovered a relationship, whereas our investigation found none. This difference could be a reference to the occupational categories, as all of the participants in this study are employed by a dentistry school. In comparison, the study by Farooqui *et al.* includes distinct groups such as unemployed, students, homemakers, and retired. ^9^

By contrast, this study discovered a different association between practice scores and occupational categories. Dental nurses and administrator workers scored highest on herbal medicine practice, whereas administrator workers scored lowest on knowledge. The cause behind this is unknown, and additional research on this group may shed light on it.

Leach, 2004 found that medical nurses had a high degree of practice and a positive attitude toward alternative medicines. According to which dental nurses had the highest practice score for herbal medicine, this finding may corroborate ours. At the same time, it contradicts the point that dental nurses have demonstrated a low level of practice with herbal medicines. This conflicting statement could be referring to the difference between dental and medical nurses, as well as the fact that herbal medicine is a subcategory of alternative medicine. Thus, high level of practice may be related to many forms of complementary and alternative medicine. Additionally, oral health applications are separate from those for other medical conditions. ^27^

## Conclusions

The dental staff at the School of Dental Sciences has a moderate level of knowledge and a poor level of practice in herbal medicines for oral health. There was a significant association between age and knowledge level, but not between age and practice. The relationship between knowledge and practice and sex is not statistically significant. The specific occupation of dental staff, on the other hand, affected the level of practice. There was a significant correlation between the level of knowledge and practice about herbal medicines used for oral health.

### Recommendations

Further research is needed to determine the Malaysian population’s knowledge and practice of herbs for oral health, as no studies on this subject have been conducted according to our understanding. Most studies focused on using herbal medicines during pregnancy, cancer treatment, and general health. Within the School of Dental Sciences at USM, group discussions and lectures on herbal medicines related to oral health may help to increase staff knowledge.

## Data Availability

Figshare: Knowledge and Practice of Herbal Medicine on Oral Health among Dental Personnel in Malaysia, https://doi.org/10.6084/m9.figshare.21834972.v1. ^28^ This project contains the following underlying data:
• Herbal Medicine for OH Data (Spreadsheet for participants answers) This project contains the following extended data:
• KP regarding Herbal Medicine for Oral Health questionnaire (The questionnaire used in the study) Data are available under the terms of the Creative Commons Zero “No rights reserved” data waiver (CC0 1.0 Public domain dedication). Access to this dataset requires registration with an IEEE account, which is free.
