# Supplementary material for: Knowledge and Practice of Herbal Medicine on Oral Health among Dental Personnel in Malaysia
Source: F1000Res. 2023 Mar 14;12:283. [Version 1] doi: 10.12688/f1000research.129865.1 (PMC12423618; doi:10.12688/f1000research.129865.1)
Supplement: Peer review history

# Reviewer report: Reviewer response for version 1

**DOI:** 10.5256/f1000research.142581.r410117

**Published:** 2025-09-24

**Authors:** Maha Sh Mahmood

**Affiliations:** 1 University of Baghdad, Baghdad, Baghdad Governorate, Iraq

**Reviewer Report**

**Title:** Knowledge and Practice of Herbal Medicine on Oral Health among Dental Personnel in Malaysia

**Authors:** Abedelmalek Kalefh Tabnjh et al.

**Letter to the Authors**

 Dear Authors,

 Thank you for submitting your manuscript *“Knowledge and Practice of Herbal Medicine on Oral Health among Dental Personnel in Malaysia”* to **F1000Research**. I found your study to be an important and timely contribution to the understanding of complementary and alternative medicine in dentistry, particularly within the Malaysian context.

 Your methodology is sound, and the findings—especially the gap between knowledge and practice, and the occupational differences in practice—are both novel and relevant. However, I believe the manuscript would be strengthened by some revisions:

**Major Comments**

1. **Introduction**
  -
    - The introduction provides good background but would benefit from a stronger justification of the study’s significance. For instance, the link between the high use of herbal/alternative medicine in Malaysia and the need for awareness among dental personnel could be emphasized further.
2. **Methods**
  -
    - The sampling method is well explained, but the inclusion criteria should clearly define employment duration at USM (e.g., ≥6 months) to avoid bias from very recent hires with limited knowledge.
    - More details on questionnaire validation (content and construct validity and reliability) should be included to strengthen confidence in the implement.
3. **Results**
  -
    - The results are clear and supported with tables. However, some tables (e.g., Table 3 and Table 5) are dense; summarizing key findings in the text more concisely would improve readability.
    - Missing data handling (age and income) should be explained more thoroughly.
4. **Discussion**
  -
    - The discussion appropriately compares findings with other studies. However, it could explain deeper reasons for low practice despite moderate knowledge , for example, possible reliance on conventional dentistry.
    - The occupational differences in practice deserve more detailed exploration, as this is a novel and interesting finding.
    - Limitations should be expanded (single-center, predominantly Malay sample, self-reported data, possible social desirability bias).
5. **Conclusions and Recommendations**
  -
    - The conclusions are consistent with results, but the practical implications should be more strongly pronounced e.g., implications for training curricula, continuing education, and policy on integrating herbal medicine into oral health care.

**Minor Comments**

- Language and grammar are generally fine, but some sentences could be simplified for clarity. Example: *“The results, from the other hand…”* should be revised to *“On the other hand, the results revealed…”*in the abstract section.
- Tables should be checked for formatting consistency (e.g., Table 8 alignment).
- Ensure all references are updated and formatted according to journal style. Some citations (e.g., Azriani 2007 thesis reference number 13  ) could use clearer referencing details.

**Recommendation**

**Minor to Moderate Revisions** – The manuscript is scientifically sound and merits indexing after the authors address the points above, particularly clarifying methodology, strengthening discussion, and refining presentation.

 Overall, this is a valuable manuscript that should be of interest to the F1000Research readership. I encourage you to revise accordingly and resubmit.

 Best regards,

 Prof. Maha Shukri Mahmood

 College of Dentistry, University of Baghdad

 Reviewer for F1000Research

Is the work clearly and accurately presented and does it cite the current literature?

Yes

If applicable, is the statistical analysis and its interpretation appropriate?

Yes

Are all the source data underlying the results available to ensure full reproducibility?

Yes

Is the study design appropriate and is the work technically sound?

Yes

Are the conclusions drawn adequately supported by the results?

Yes

Are sufficient details of methods and analysis provided to allow replication by others?

Yes

Reviewer Expertise:

Periodontics

I confirm that I have read this submission and believe that I have an appropriate level of expertise to confirm that it is of an acceptable scientific standard, however I have significant reservations, as outlined above.

# Reviewer report: Reviewer response for version 1

**DOI:** 10.5256/f1000research.142581.r410110

**Published:** 2025-09-10

**Authors:** Fiki Muhammad Ridho

**Affiliations:** 1 Dental Profession Program, Faculty of Dental Medicine, Universitas Airlangga, Surabaya, Indonesia

In general, this study is good and makes a meaningful contribution to the literature on oral health. However, there are several points that could be considered in order to further strengthen the manuscript:

 1. Abstract. There appears to be an inconsistency between the Abstract and the Results section. In the Abstract it is stated that “…there was no significant relationship between knowledge and practice scores and age or gender.”, whereas in the Results a significant association between knowledge score and age is reported (p = 0.018). Please recheck, correct, and align the Abstract with the Results.

 2. Introduction. The background is well presented, but there is general claim, for example the perception of Malaysians regarding the safety of herbal products (paragraph 7). It is not clear whether this represents Malaysians in general or only a specific population in certain areas. It is recommended to revise such claims to be specific to the study sample so as not to imply unsupported generalization. In addition, the importance of conducting this study could be further emphasized.

 3. Methods. I cannot comment on the methodological/statistical aspects of the study. A qualified statistician is required for that part.

 4. Discussion. The discussion is generally clear and well written. Nevertheless, the authors are encouraged to add a section on the practical implications of the findings, for instance how the results could be utilized in dental education and practice. The authors are also advised to expand the limitations section, so that the limitations of the study are described in a more comprehensive manner.

 Overall, this is a relevant and interesting study. I wish you all the best. Thank you.

Is the work clearly and accurately presented and does it cite the current literature?

Yes

If applicable, is the statistical analysis and its interpretation appropriate?

I cannot comment. A qualified statistician is required.

Are all the source data underlying the results available to ensure full reproducibility?

Yes

Is the study design appropriate and is the work technically sound?

Yes

Are the conclusions drawn adequately supported by the results?

Yes

Are sufficient details of methods and analysis provided to allow replication by others?

Yes

Reviewer Expertise:

Dental medicine, Herbal medicine

I confirm that I have read this submission and believe that I have an appropriate level of expertise to confirm that it is of an acceptable scientific standard.

# Reviewer report: Reviewer response for version 1

**DOI:** 10.5256/f1000research.142581.r277906

**Published:** 2024-06-11

**Authors:** Muhd Firdaus Che Musa

**Affiliations:** 1 Department Of Paediatric Dentistry And DPH Kulliyyah Of Dentistry, International Islamic University Malaysia, Kuantan, Malaysia

Dear Authors, 

 The research work is generally excellent. Well done!

 The article could be enhanced with the following general comments:

 1. The introduction section could benefit from additional justification for the importance of the research, particularly in light of findings from a previous study indicating that over half of the Malaysian participants resorted to alternative medicine to manage orofacial pain.

 2. While it is acknowledged that there is limited evidence on this research topic, it would be beneficial to also explore the relationship between knowledge and practice by conducting a comprehensive literature review across various health fields.

 3. It would be beneficial to define the inclusion criteria as follows: USM employees who have been employed for a specific duration.

 Well done. Wish you all the best.

 Thank you.

Is the work clearly and accurately presented and does it cite the current literature?

Yes

If applicable, is the statistical analysis and its interpretation appropriate?

Yes

Are all the source data underlying the results available to ensure full reproducibility?

Yes

Is the study design appropriate and is the work technically sound?

Yes

Are the conclusions drawn adequately supported by the results?

Yes

Are sufficient details of methods and analysis provided to allow replication by others?

Yes

Reviewer Expertise:

Preventive and Community Dentistry

I confirm that I have read this submission and believe that I have an appropriate level of expertise to confirm that it is of an acceptable scientific standard.
